# Effective polyclonal antibodies against the virulence-associated protein D (VapD) of *Helicobacter pylori*, obtained from recombinant VapD

**DOI:** 10.1371/journal.pone.0321455

**Published:** 2025-04-16

**Authors:** Alejandro Flores-Alanis, Gabriela Delgado, Carlos Santiago-Olivares, Víctor Manuel Luna-Pineda, Armando Cruz-Rangel, Denilson Guerrero-Mejía, María Luisa Escobar-Sánchez, Nayeli Torres-Ramírez, Rosario Morales-Espinosa

**Affiliations:** 1 Departamento de Microbiología y Parasitología, Facultad de Medicina, Universidad Nacional Autónoma de México, Mexico City, Mexico; 2 Laboratorio de Investigación en Patógenos Respiratorios y Producción de Biológicos, Hospital Infantil de México “Federico Gómez”, Mexico City, Mexico; 3 Laboratorio de Bioquímica de Enfermedades Crónicas, Instituto Nacional de Medicina Genómica, Mexico City, Mexico; 4 Laboratorio de Inmunobiología y Diagnóstico Molecular, Facultad de Ciencias Químico Biológicas, Universidad Autónoma de Guerrero, Guerrero, Mexico; 5 Departamento de Biología Celular, Facultad de Ciencias, Universidad Nacional Autónoma de México, Mexico City, Mexico; Universidad Nacional de la Plata, ARGENTINA

## Abstract

*Helicobacter pylori* is a microorganism associated with serious gastric pathologies. This bacterium presents specific genes that encode for different virulence factors associated with the development of gastric disease. The VapD protein has rarely been studied, although it has been previously demonstrated its participation in the protection of *Helicobacter pylori* within gastric cells. In the present work, we document the protocols developed to generate the VapD recombinant protein and the subsequent production of polyclonal antibodies. Our research group faced several problems throughout the trials; however, all of them were successfully solved.

## Introduction

Virulence-associate protein D (VapD) from *Helicobacter pylori* was first structurally and biochemically characterized 12 years ago [1]. It is a small protein of 11.2 kDa with purine-specific endoribonuclease activity related to the Cas2 family from the CRISPR/Cas system-associate proteins. Nonetheless, there are no studies that documented the potential role of this protein in *H. pylori* pathogenesis. VapD, however, has been studied in other microorganisms, including *Rhodococcus equi* and *Haemophilus influenzae*, suggesting that *vapD* may protect these bacteria from respiratory bursts within macrophages, or facilitate persistence in the respiratory epithelial cells microenvironment, respectively [2–4]. On the other hand, the *vapD* gene has been found to be overexpressed during the biofilm formation of pathogenic strains of *Xylella fastidiosa*; suggesting a role in bacterial virulence [5].

We reported that the transcription of the *vapD* gene in *H. pylori* when observed in adenocarcinoma gastric cells (AGS) [6]. In this microenvironment, this bacterium adopted a coccoid form, but still metabolically active. This fact was demonstrated through real-time PCR [6]. Moreover, it has been also observed the expression of the *vapD* in gastric biopsies of patients with atrophic chronic gastritis, follicular gastritis, peptic ulcers, and gastric adenocarcinoma [6]. These findings suggest the expression of the VapD protein, and its possible significant in the protection and/or persistence of *H. pylori* within gastric epithelial cells. Consequently, we believe that studying the role of VapD in the pathogenesis of chronic *H. pylori* infection is crucial to the understanding of the development of chronic inflammation and oncogenesis.

In the present study, we developed reproducible protocols that enable the cloning, expression and purification of recombinant VapD (rVapD) from *H. pylori*, as well as the production of polyclonal antibodies in mice. These antibodies were effective for *in vivo* immunolocalization and *in vitro* immunoprecipitation of VapD. Throughout the various assays, we encountered and successfully solved several technical challenges.

## Materials and methods

The protocol described in this peer-reviewed article has been previously published in the server protocols.io, (dx.doi.org/10.17504/protocols.io.dm6gpz251lzp/v1) which is included for printing purposes in the Supplementary Material (S1 File).

### Ethics statement

The protocol was approved by the International Committee for the Care and Use of Laboratory Animals (CICUAL) of the Autonomous University of Guerrero with office number CICUAL-03/2023.

## Expected results

**Fig 1:**
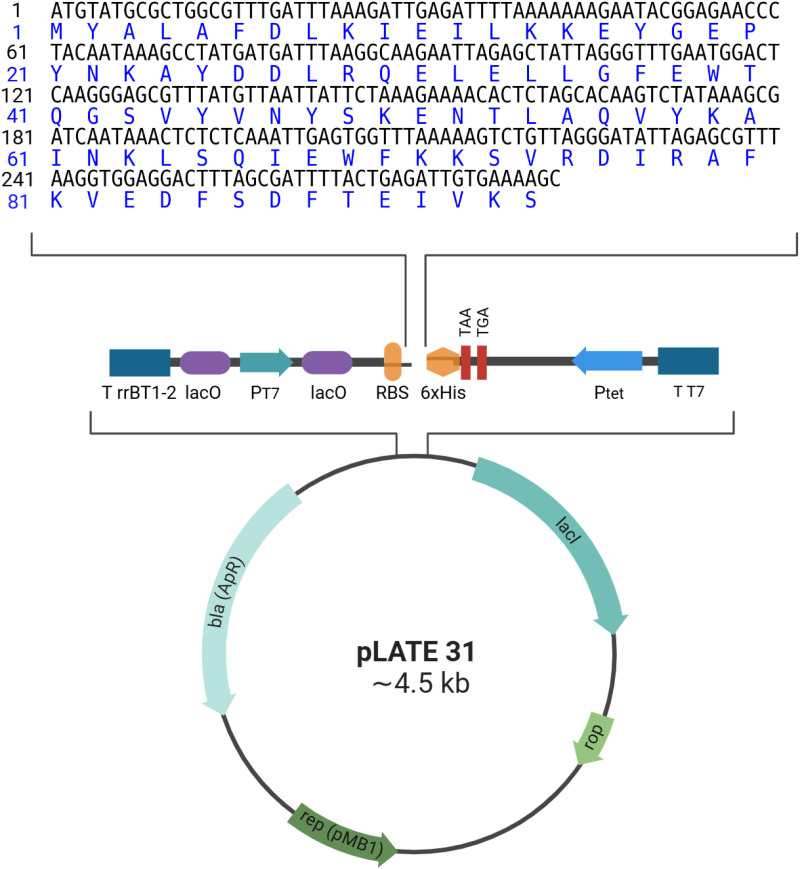
Construction of pLATE31-vapD-6xHis. The *vapD* gene lacking a stop codon was cloned into a pLATE31 vector. This expression vector presents a six histidine coding sequence (6xHis), which allows the expression of the protein with a C-terminal 6xHis-tag. Additionally, the vector presents the following characteristics: a β-lactamase gene (*bla*) conferring resistance to ampicillin (AP^R^); an origin of replication (rep [pMB1]); the rop protein gene, which regulates the number of copies of the plasmid; the lac repressor (lacl), which ensures a strict control of the basal expression of the T7 RNA polymerase promoter (PT7); the T rrnBT1-2 transcription terminator, which prevents basal gene expression from vector derived promoter-like elements; the lacO operator, which ensures a strict control of gene expression; a ribosome binding site (RBS); Ptet, a promoter reduces basal expression from the PT7; the T7 terminator (TT7), which terminates transcription from the PT7. The vector was created in BioRender (https://BioRender.com).

The *vapD* gene from *H. pylori* strain 22695 was identified through end-point PCR, resulting in a 282 bp amplicon. This amplicon was subsequently cloned into the pLATE31 expression vector and transformed into *E. coli* Rosetta (DE3). The colonies were screened by PCR, and the plasmids from positive colonies were verified through Sanger sequencing. The *vapD* gene was located in an open-read frame with a six histidine tag at the 3’ end (Fig 1).

**Fig 2:**
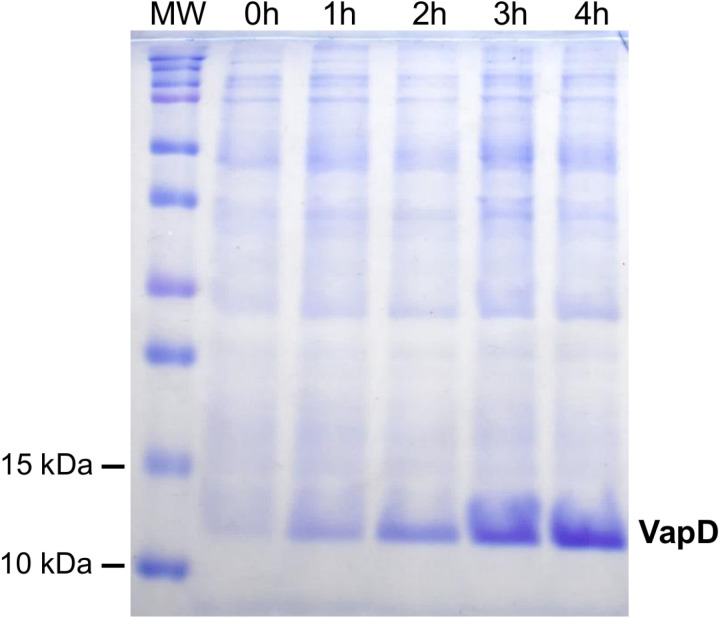
Expression of rVapD in *E. coli* Rosetta (DE3). Aliquots of 20 µl of culture were collected at 0, 1, 2, 3, and 4 h after the addition of 0.1mM IPTG to verify the correct expression of rVapD by SDS-PAGE. MW, molecular weight.

**Fig 3:**
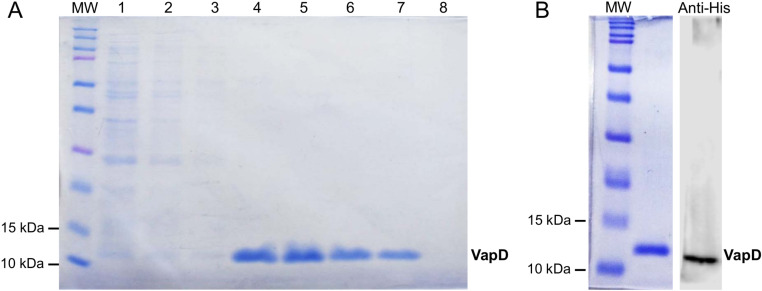
Purification and identification of rVapD by IMAC. A. Recuperation of rVapD. Aliquots of 20 µl from each elution were separated by SDS-PAGE. Lane 1: flow-through of the *E. coli* Rosetta (DE3) lysate after the His-tag column; lanes: 2-3, washes with buffer containing 50 mM imidazole; lanes: 4-8, rVapD eluted from the His-tag column with buffer containing 325 mM imidazole. B. rVapD was concentrated after IMAC and confirmed by Western blotting anti-His using 1 µg of rVapD. MW, molecular weight.

*E. coli* Rosetta (DE3) carrying the pLATE31-*vapD*-6xHis construct was induced with 0.1 mM IPTG.The rVapD protein was observed at approximately 12 kDa (Fig 2). No difference in protein expression was observed with different IPTG concentrations (0.5 and 1.0 mM). The His-tagged rVapD was purified through immobilized metal affinity chromatography (IMAC) (Fig 3A). The protein was then concentrated, and its identity was confirmed by Western blotting with an anti-His antibody (Fig 3B).

**Fig 4:**
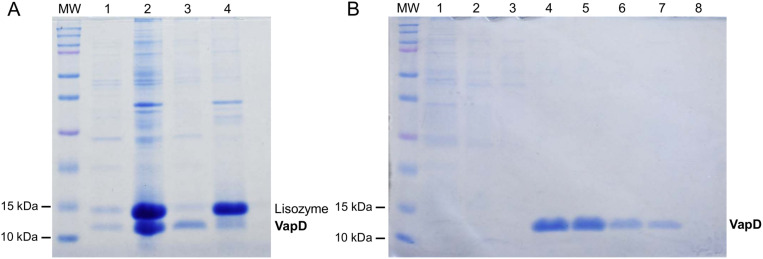
Use of non-denaturing detergents in rVapD purification. A. Recovery of rVapD. Lanes 1 and 2: soluble (20 µg) and insoluble (2 µl) fractions, respectively, from *E. coli* Rosetta (DE3) lysate overexpressing rVapD with lysozyme method. Lanes 3 and 4: soluble (20 µg) and insoluble (2 µl) fractions from *E. coli* Rosetta (DE3) lysate overexpressing rVapD with lysozyme and non-denaturing method, respectively. B. Purification of rVapD by IMAC after non-denaturing method. Aliquots of 20 µl from each elution were separated by SDS-PAGE. Lane 1: flow-through of the *E. coli* Rosetta (DE3) lysate after the His-tag column; lanes: 2-3, washes with buffer containing 50 mM imidazole; lanes: 4-8, rVapD eluted from the His-tag column with buffer containing 325 mM imidazole. MW, molecular weight.

We observed that when bacterial lysis was performed with lysozyme, rVapD was located in the insoluble fraction (Fig 4A). However, non-denaturing methods are available for recovering soluble proteins from inclusion bodies by addition a mix of detergents such as CHAPS, Triton X-100 or sarkosyl. Studies have shown that combining these detergents yields better results than using them individually [7,8]. In these studies, optimal results were achieved with 1–2% sarkosyl, 2–4% Triton X-100 and 20–40 mM CHAPS. The authors initially solubilized the insoluble pellet with sarkosyl for 6–12 h after centrifugation; subsequently, to increase the efficiency of binding to chromatography, Triton X-100 and CHAPS were added as well.

In this work, we reduced the concentration of sarkosyl to 0.7% to prevent the protein extract from becoming too viscous. We employed a mix of the three detergents simultaneously after the bacteria pellet was resuspended, as previously reported [9]. The results were similar: the purification ratio between our method and the previously reported detergent concentrations [7] was 1:0.96, suggesting that the modifications did not alter the purification. Furthermore, we observed that the yield of rVapD was lower when it was purified without the use of detergents (0.66 mg/ml) than when it was purified with non-denaturing detergents (1.7 mg/ml). Our method did not affect the purification efficiency of rVapD (Fig 4B).

A previous study reported complications when obtaining stable *H. pylori* rVapD protein fused to a His-tag, as it precipitated at concentrations above 25 µM. The authors addressed this issue by fusing VapD with the streptococcal protein G (GB1) [1]. In the present work, we observed that after IMAC purification, rVapD remained stable at concentrations up to 150 µM for one month at 4°C. This fact did not represent a problem for our objectives; however, it should be considered for further studies.

**Fig 5:**
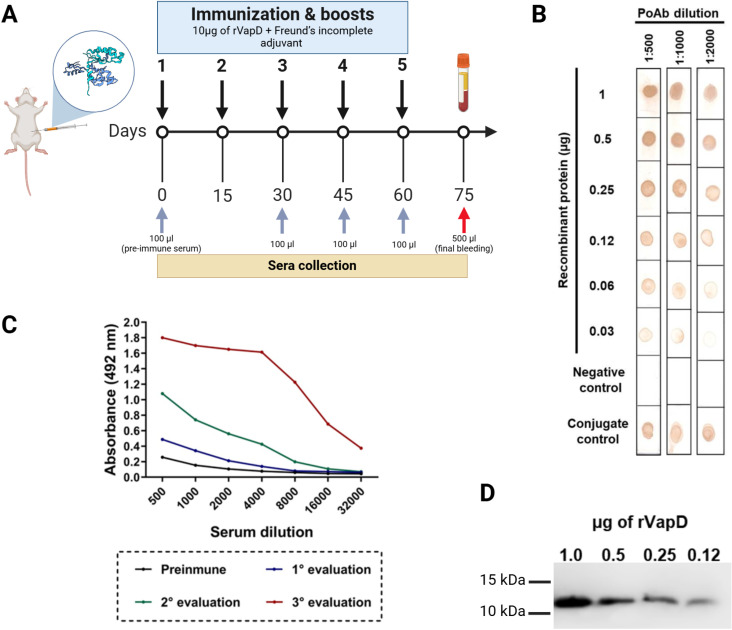
Production and testing of polyclonal antibodies against rVapD. A. Representation of the immunization schedule. B. Absorbance values of the indirect ELISA with serum tested at 30, 45 and 60 days (gray arrows in A), rVapD was used at a final concentration of 100 ng. C. Dot blotting test of the final bleeding (red arrow in A) using three dilutions of the serum (1:500, 1:1,000, and 1:2,000) and a serially two-fold diluted concentration of rVapD (1 to 0.03 µg). D. Western blotting of the serum at a dilution of 1:2,000 against a serially two-fold diluted concentration of rVapD (1 to 0.12 µg). Fig 5A was created in BioRender (https://BioRender.com).

Although VapD is a small protein (11.2 kDa), we found that the combination of rVapD with Freund’s adjuvant was sufficient to induce an immune response, as depicted in Fig 5A. The result of the indirect ELISA demonstrated that immunization and booster doses increased the immune response over time; at the end of the immunization schedule, the highest titers were observed at dilutions between 1:500 and 1:4,000 (Fig 5B). The polyclonal serum from the final bleeding of the mice was tested against various concentrations of rVapD; dot blotting revealed that the serum recognized up to 30 ng of rVapD at dilutions of 1:500 and 1:1,000, whereas at a dilution of 1:2,000, rVapD was detected at a concentration of 60 ng (Fig 5C). Finally, the serum was tested by Western blot against the same rVapD concentrations used in the dot blot, with a 1:2,000 dilution (Fig 5D).

**Fig 6:**
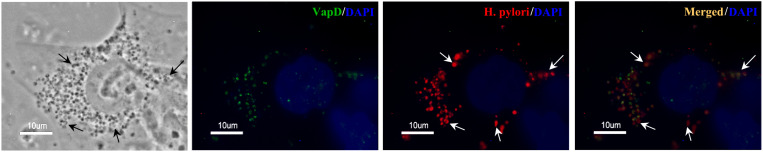
Detection of VapD from *H. pylori in vivo* by immunofluorescence assay. *H pylori* was observed in AGC cells at 48 h post-inoculation. The expression of VapD coincided with the presence of the bacterium. The arrows point to vesicles where *H. pylori* is internalized.

To demonstrate the *in vivo* expression of the VapD protein, we performed an immunofluorescence assay in co-cultures of AGS cells and *H. pylori* strain 26695 at 48 h post-inoculation. The co-cultures were incubated with anti-vapD (green) and anti-*H. pylori* (red) antibodies. The presence of VapD corresponded with the localization of intracellular bacteria (Fig 6). Furthermore, we observed multiple vesicles, some of which contained bacteria (arrows in Fig 6).

**Fig 7:**
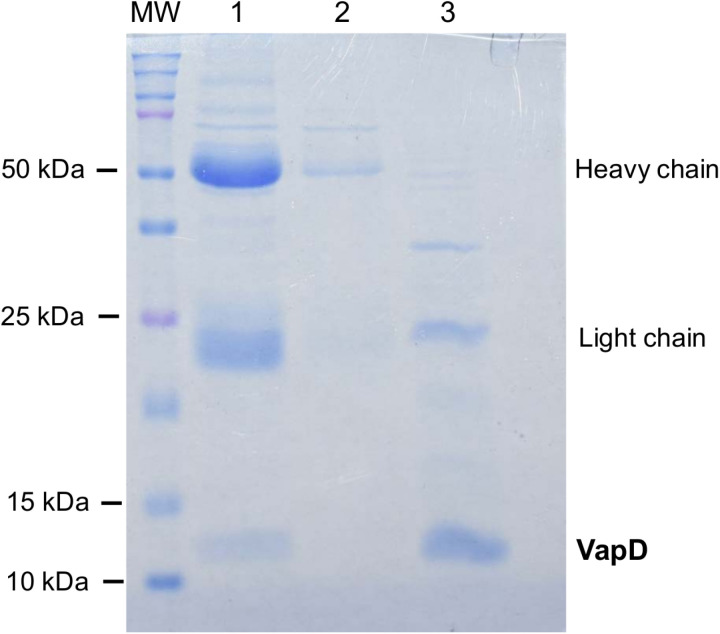
Immunoprecipitation of rVapD. Lane 1: mouse serum (5 µl) + bacterial lysate (20 µl); lane 2: mouse pre-immune serum (5 µl) + bacterial lysate (20 µl); lane 3: lysate of Rosetta (DE3) cells overexpressing rVapD (20 µl). MW, molecular weight.

Anti-VapD polyclonal antibodies were also tested through an immunoprecipitation (IP) assay. IP efficiency was evaluated using lysates from Rosetta (DE3) cells overexpressing rVapD, and the antibodies successfully immunoprecipitated rVapD (Fig 7).

This work describes the protocols for the production, expression, and purification of *H. pylori* rVapD, as well as the production of its antibodies. The results indicate that the polyclonal antibodies successfully recognized both the quaternary and linear structures of VapD. The immunolocalization and IP results revealed that the polyclonal serum recognized the structural epitopes, whereas the Western blot result demonstrated the recognition of linear epitopes. These findings open the possibility of selecting monoclonal antibodies using the hybridoma method to identify antibodies on the basis of specific applications for further investigation. This is the first study to report the production of specific antibodies against VapD.

## Supporting information

S1 File Step-by-step protocol, also available on protocols.io. (PDF)

S2 Raw Images Raw images. (PDF)

S3 File Values used to build the graph in Fig 5C. (XLSX)
