# Supplementary material for: Effective polyclonal antibodies against the virulence-associated protein D (VapD) of Helicobacter pylori, obtained from recombinant VapD
Source: PLoS One. 2025 Apr 16;20(4):e0321455. doi: 10.1371/journal.pone.0321455 (PMC12002528; doi:10.1371/journal.pone.0321455)
Supplement: S1 File — (PDF) [file pone.0321455.s001.pdf]

# Effective polyclonal antibodies against the virulence-associated protein D (vapD) of *Helicobacter pylori*, obtained from recombinant VapD

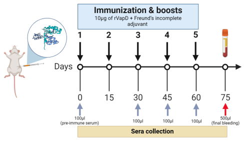


RESERVED DOI:

**10.17504/protocols.io.dm6gpz251lzp/v1**

Alejandro Flores-Alanis[1], Gabriela Delgado[1], Carlos Santiago-Olivares[1], Víctor Manuel Luna-Pineda[2], Armando Cruz-Rangel[3], Denilson Guerrero-Mejía[4], María Luisa Escobar-Sánchez[5], Nayeli Torres-Ramírez[5], Rosario Morales-Espinosa[1]

[1]Departamento de Microbiología y Parasitología, Facultad de Medicina, Universidad Nacional Autónoma de México, Mexico City, Mexico;
[2]Laboratorio de Investigación en Patógenos Respiratorios y Producción de Biológicos, Hospital Infantil de México "Federico Gómez", Mexico City, Mexico;
[3]Laboratorio de Bioquímica de Enfermedades Crónicas, Instituto Nacional de Medicina Genómica, Mexico City, Mexico;
[4]Laboratorio de Inmunobiología y Diagnóstico Molecular, Facultad de Ciencias Químico Biológicas, Universidad Autónoma de Guerrero, Guerrero, Mexico;
[5]Departamento de Biología Celular, Facultad de Ciencias, Universidad Nacional Autónoma de México, Mexico City, Mexico

Alejandro Flores-Alanis
UNAM

**Protocol Info:** Alejandro Flores-Alanis, Gabriela Delgado, Carlos Santiago-Olivares, Víctor Manuel Luna-Pineda, Armando Cruz-Rangel, Denilson Guerrero-Mejía, María Luisa Escobar-Sánchez, Nayeli Torres-Ramírez, Rosario Morales-Espinosa . Effective polyclonal antibodies against the virulence-associated protein D (vapD) of Helicobacter pylori, obtained from recombinant VapD. **protocols.io https://protocols.io/view/effective-polyclonal-antibodies-against-the-virule-dixh4fj6**

**Created:** July 09, 2024

**Last Modified:** February 04, 2025

**Protocol Integer ID:** 105161

**Keywords:** Helicobacter pylori, virulence-associated protein D (VapD), recombinant protein, protein purification, polyclonal antibodies

**Funders Acknowledgements:**
**DGAPA-PAPIIT**
**Grant ID: IN213921**
**CONAHCYT**
**Grant ID: CF-2023-G-919**

# Abstract

*Helicobacter pylori* is a microorganism associated with serious gastric pathologies. It has different genes that encode for different virulence factors associated with the development of the disease. The VapD protein has rarely been studied, although it has been shown that it participates in the protection of *Helicobacter pylori* within gastric cells. In the present work, we document the protocols used to produce the VapD recombinant protein and the production of polyclonal antibodies. Our research group faced different problems throughout the trials; however, all of them were successfully resolved.

## Materials

**Materials and reagents:**

1. Synthetic primers for PCR amplification
2. Agarose gels
3. LB medium
4. LB agar plates containing ampicillin (100 µg/ml)
5. Blood agar plates
6. Electroporation cuvettes of 0.1 cm
7. 15 ml tube with a screw cap
8. 250 ml Erlenmeyer flask
9. Isopropyl-thio-β-D-galactopyranoside (IPTG)
10. EDTA-free protease inhibitor cocktail
11. Phenylmethylsulfonyl fluoride (PMSF)
12. 20% SDS-PAGE gels
13. Chromatography column
14. Skimmed milk powder
15. 96-well high binding plate
16. Nitrocellulose membrane
17. PVDF membrane
18. Dulbecco´s Modified Eagle's Medium (DMEM)
19. Fetal bovine serum
20. 60 mm crystal polystyrene Petri dishes
21. 120 mm crystal polystyrene Petri dishes
22. Coverslips
23. HisPur™ Ni-NTA Resin (Thermo Fisher Scientific, Massachusetts, USA)
24. HiPrep 26/10 Desalting (Cytiva™, Massachusetts, USA)
25. Freund’s incomplete adjuvant (Sigma-Aldrich, Massachusetts, USA)
26. Chemiluminescence reagent SuperSignal™ West Pico PLUS (Thermo Fisher Scientific, Massachusetts)
27. Antifade Mounting Medium (Vectashield®, California, USA)
28. Quick Start™ Bradford 1X dye reagent (Bio-Rad, California, USA)
29. Protein A/G agarose beads (Santa Cruz Biotechnology Inc. California, USA)
30. Coomassie® Brilliant blue R 250 (Merck, New Jersey, USA)

**Kits:**

1. Platinum™ Taq DNA Polymerase High Fidelity **Thermo Fisher Catalog #**11304011 .
2. MinElute Gel extraction kit **Qiagen Catalog #**28604 .
3. aLICator LIC Cloning and Expression Kit 3 (C-terminal His-tag) **Thermo Fisher Catalog #**K1261 .
4. PureDireX, Plasmid miniPREP Kit **Bio-Helix Catalog #**PDP01-0100 .

**Bacterial strains and cell culture:**

1. Competent *Escherichia coli* DH5α
2. Competent *Escherichia coli* Rosetta (DE3)
3. *Helicobacter pylori* strain 26695 (ATCC 700392)
4. Adenocarcinoma cell line (AGS) (ATCC CRL-1739)

**Antibodies:**

1. Antibody anti-mouse H+L coupled to horseradish peroxidase (Santa Cruz Biotechnology Inc. California, USA)
2. Antibody anti-*H. pylori* developed in rabbit (Biocare Medical, California, USA)
3. Antibody anti-mouse Alexa Fluor 594 (Life Technologies, Eugene, OR)
4. Antibody anti-rabbit Alexa Fluor 488 (Life Technologies, Eugene, OR)

**Buffers and solutions:**

- TE buffer

| A | B |
|---|---|
| Tris HCl, pH 8.0 | 10 mM |
| EDTA | 1 mM |

- BMR4× buffer

| A | B |
|---|---|
| Tris, pH 6.8 | 320 mM |
| SDS | 8 % w/v |
| Glycerol | 40% v/v |
| Bromophenol blue | 0.008 % w/v |
| 2-Mercaptoethanol | 21 % v/v |

- SDS-PAGE running buffer 10×

| A | B |
|---|---|
| Tris | 250 mM |
| Glycine | 2.5 M |
| SDS | 1 % w/v |

- Coomassie brilliant blue solution

| A | B |
|---|---|
| Coomassie brilliant blue R-250 | 0.5 % w/v |
| Methanol | 50 % v/v |
| acetic acid | 7 % v/v |

- Lysis buffer

| A | B |
|---|---|
| Tris HCl, pH 8.0 | 50 mM |
| Glycerol | 5 % v/v |
| 2-Mercaptoethanol | 1 mM |
| EDTA | 0.1 mM |

- Equilibration buffer

| A | B |
|---|---|
| Tris HCl, pH 8.0 | 50 mM |
| NaCl | 150 mM |
| EDTA | 0.1 mM |
| DTT | 1 mM |

- Wash buffer (equilibration buffer containing 50 mM imidazole)
- Elution buffer (equilibration buffer containing 325 mM imidazole)
- PBS 10×

| A | B |
|---|---|
| NaCl | 1.37 M |
| KCl | 27 mM |
| Na2HPO4 | 100 mM |
| KH2PO4 | 18 mM |
| pH | 7.4 |

- Carbonate-bicarbonate buffer

| A | B |
|---|---|
| Na2CO3 | 4 mM |
| NaHCO3 | 46 mM |
| pH | 9.2 |

- PBT (PBS 1× containing 0.1% v/v Tween 20)
- Phosphate-citrate buffer

| A | B |
|---|---|
| Na2HPO4 | 51 mM |
| Citric acid [$HOC(CH_2CO_2H)_2$] | 24 mM |

- OPD developer solution

| A | B |
|---|---|
| Phosphate-citrate buffer, pH 5.0 | 50 mM |
| Ortho-102 phenylenediamine (OPD) | 0.4 mg/ml |
| Hydrogen peroxide (H2O2) | 30% v/v |
| Add the H2O2 immediately prior the use | |

- DAB developer solution (PBS 1× containing 0.5 mg/ml of 3,3'-diaminobenzidine (DAB) and 30% v/v $H_2O_2$). Make the solution just before use.
- Western blot transfer buffer (SDS-PAGE running buffer 1× 10% v/v, methanol 20% v/v)
- Fixative solution (PBS 1× pH 7.2, 2% v/v paraformaldehyde)
- Permeabilizing solution (PBS 1×  pH 7.2, Triton X-100 0.5% v/v)

**Equipment:**

1. MJ Mini™ 113 PCR thermal cycler (Bio-Rad, California, USA)
2. Spectrophotometer NanoDrop™ 114 (Thermo Fisher Scientific, Massachusetts, USA).
3. Horizontal electrophoresis chamber (Bio-Rad, California, USA. No cat.1704482).
4. Ultra-violet (UV) transilluminator (Analytikjena, Jena, Germany)
5. Electroporator Gene Pulser Xcell Microbial system (Bio-Rad, California, USA. No cat.1652666)
6. Incubator at 37 ºC (Thermo Fisher Scientific, Massachusetts, USA)
7. Shaker incubator at 37 ºC (mrc Laboratory Instruments, Harlow, UK)
8. Microplate reader spectrophotometer (TECAN, Männedorf, Switzerland)
9. SDS-PAGE electrophoresis chamber (Bio-Rad, California, USA. No cat.1652666)
10. Sonic dismembrator Fisherbrand™ 126 (Thermo Fisher Scientific, Massachusetts, USA)
11. Refrigerated ultracentrifuge (Thermo Fisher Scientific, Massachusetts, USA)
12. FPLC (Cytiva™ 129 , Massachusetts, USA)
13. Tank blotting system (Bio-Rad, California, USA)
14. C-DiGit® 131 blot scanner (LI-COR, Nebraska, USA)
15. Nikon Eclipse E600 epifluorescence microscope (Nikon, NY, USA)

# Before start

The reagents, vendors and manufacturers used can be replaced by similar ones.

## Construction of the expression plasmid and bacteria transformation

8h 28m

1 The *vapD* gene was amplified by PCR. The PCR mixture was prepared as follows:

| A | B |
|---|---|
| High fidelity buffer | 1X |
| MgSO4 | 1.5 mM |
| dNTPs | 0.6 mM |
| Primer VapDcter forward 5'-AGA AGG AGA TAT AAC TAT GTA TGC GCT GGC GTT TGA TCT-3' | 0.5 µM |
| Primer VapDcter reverse 5'-GTG GTG GTG ATG GTG ATG GCC GCT TTT CAC AAT TTC GGT-3' | 0.5 µM |
| Platinum ® 140 Taq DNA polymerase High Fidelity | 1U |
| DNA (the plasmid construction pCR 2.1-vapD was used as template) | 1 µl |
| Water for final volume of 25 µl | X µl |

2 Program the PCR thermal cycler with the following conditions: denaturation at 94 °C for 00:05:00 , followed by 30 cycles of denaturation at 94 °C for 00:01:00 , annealing at 52 °C for 00:01:00 and extension at 72 °C for 00:01:00 ; and a final extension at 72 °C for 00:10:00 .

18m

3 Run the PCR product by electrophoresis on agarose gel and visualize it on an UV transilluminator.

4 Purify the PCR product from agarose gel electrophoresis using the MinElute Gel Extraction Kit following the manufacturer's instructions.

5 Quantify the PCR product by measuring its absorbance at 260/280 nm using a spectrophotometer.

6 Generate the VapD-6xHis fusion vector using the aLICator™ LIC Cloning and Expression System Kit 3:

6.1 Add 2 µL of 5× LIC buffer, 1 µL of T4 DNA polymerase, 20 ng of purified PCR product, and enough water to adjust the final volume to 9 µL . Vortex briefly.

6.2 Incubate the reaction mixture at Room temperature for 00:05:00 .

5m

6.3 Stop the reaction by adding 0.6 µL of [M] 0.5 Molarity (M) EDTA and mix well.

6.4 For annealing, add 1 µL of pLATE31 LIC-ready vector ( 60 ng ) to the reaction. Vortex briefly.

6.5 Incubate the annealing reaction at Room temperature for 00:05:00 .

5m

**Note**

We attempted to clone *vapD* gene into various expression vectors, including pGEx-4T-2, pET-28b(+), and pET30-GBFusion1, but were not successful.

7 Transform by electroporation 1 µL - 6 µL of the annealing reaction into 50 µL of competent *E. coli* DH5α cells. Deliver the electric pulse under the following conditions: 1.8 kV, 200 mA for 0.5 milisecs.

**Note**

If an electroporator is unavailable, thermal shock can be used for transformation.

8 Spread 50 µL of the cells on an LB agar plate containing ampicillin ( 100 µg /mL ).

**Note**

If no colonies appear after transformation, centrifuge the bacterial culture at 500 xg, discard the supernatant, and add 100 µl of fresh medium. Resuspend the cell pellet thoroughly and spread it onto a plate.

9 Incubate at 37 °C Overnight .

8h

## Selection of positive *E. coli* DH5α colonies

8h

10 Pick, at least 10 colonies and perform colony PCR screening using the same conditions to amplify the vapD gene (see steps 1 and 2).

11 Inoculate 5 mL of LB medium containing ampicillin ( 100 µg /mL ) with the positive colonies.

12 Incubate the tubes at 37 °C Overnight in a shaking incubator at 200 rpm /min .

8h

## Plasmid extraction and verification of the expression vector (pLATE31-VapD-6xHis)

13 Extract the plasmid from saturated cultures using the PureDirex Plasmid miniPREP Kit, following the manufacturer's instructions.

14 Run the plasmids by electrophoresis on agarose gel.

15 Visualize the gel on an UV transilluminator.

16 Screened the positives plasmids for the vapD gene by Sanger sequencing to ensure that there are not PCR-introduced mutations, using the LIC forward and LIC reverse sequencing primers included in the aLICator™ Ligation Independent Cloning and Expression System Kit 3.

## Transformation of *E. coli* Rosetta (DE3)

8h

17 Transform by electroporation competent *E. coli* Rosetta (DE3) cells with 10 ng of the expression vector (pLATE31-VapD-6xHis). Deliver the electric pulse under the following conditions: 1.8 kV, 200 mA for 0.5milisecs.

> **Note**
>
> If an electroporator is unavailable, thermal shock can be used for transformation.
> We attempted to clone *vapD* gene into BL21 (DE3), but the attempted was unsuccessful.

18 Spread 50 µL of the cells on an LB agar plate containing ampicillin ( 100 µg /mL ).

> **Note**
>
> If no colonies appear after transformation, centrifuge the bacterial culture at 500 xg, discard the supernatant, and add 100 µl of fresh medium. Resuspend the cell pellet and spread thoroughly it onto the plate.

19 Incubate the plates at 37 °C Overnight .

8h

## Expression of the recombinant protein part 1

8h

20 Inoculate a 15 ml tube with a screw tap with 3 mL of LB medium containing ampicillin( 100 µg /mL ) with a single colony of *E. coli* Rosetta (DE3) containing the expression vector (pLATE31-VapD-6xHis).

21 Incubate the tube at 37 °C Overnight in a shaking incubator at 200 rpm /min . 8h

## Expression of the recombinant protein part 2

4h

22 Inoculate 30 mL of LB medium in a 250 ml Erlenmeyer flask with 0.5 mL of saturated overnight culture.

23 Grow the cells at 37 °C in a shaking incubator at 200 rpm /min to mild-log phase ($OD_{600nm}$ ca. 0.5).

24 Add [M] 0.1 millimolar (mM) final concentration of IPTG.

25 Incubate the culture for 04:00:00 at 37 °C with shaking. 4h

26 Transfer the culture to a 50 ml conical tube and centrifuge at 3000 rpm, 4°C , discard the supernatant. The cell pellet can be stored at -70 °C until use.

> **Note**
>
> We tested different concentrations of IPT (0.1, 0.5, and 1.0 mM) and incubation times, including overnight expression, but observed no differences. The optimal conditions were as previously mentioned.
> To verify the correct expression of the recombinant vapD protein (rVapD), take 20 µL of the culture before centrifugation, add 2.5 µL of 4× BMR buffer, and boil it for 00:03:00 . Separate the sample using SDS-PAGE and visualize it with Coomassie brilliant blue staining.

## Purification of rVapD by Immobilized Metal Affinity Chromatography (IMAC)

48m 20s

27 Resuspend the cell pellet in 10 mL of lysis buffer supplemented with EDTA-free protease inhibitor cocktail and PMSF [M] 0.2 millimolar (mM) .

28 Add 0.7% sarkosyl, 3% Triton X-100, and [M] 20 millimolar (mM) CHAPS, then mix gently for 00:15:00 at 4 °C using an end-over-end rotator. 15m

29 Lyse the cells by sonication, pulses of 00:00:10 at 70% of amplitude with intervals of 00:00:10 between each pulse, up to reach a clear cell lysate (ca. 00:10:00 ). 10m 20s

Note

If a sonic dismembrator is unavailable, the cell lysis can be performed using lysozyme as follows:

- Add 1 mg /mL of lysozyme and incubate for 01:00:00 at 4 °C with gentle agitation at end-over-end rotator.
- Add 0.7% sarkosyl, 3% Triton X-100, and [M] 20 millimolar (mM) CHAPS, then mix gently for 00:15:00 at 4 °C using an end-over-end rotator.

30 Centrifuge at 15000 x g, 00:20:00 and recover the supernatant in conical tubes of 50 ml. 20m

Note

Sarkosyl can be used at higher concentrations (1-10%) to maximize the recovery of insoluble protein. However, we observed that the addition of sarkosyl (concentration >1%) increased the viscosity of the protein solution, with higher concentrations leading to greater viscosity. If the protein solution becomes too viscous, it can be passed through sterile gauze and gently squeezed, stopping when the filtered solution begins to thicken again.

31 Add an appropriate amount of Ni-NTA resin to a chromatography column. Once the column is packed, equilibrate it with two column volumes of equilibration buffer.

32 Add the protein solution onto the column at a flow rate of 1 mL /min .

33 Wash the column with five column volumes of wash buffer.

34 Elute the rVapD protein from the resin with five volumes of elution buffer at a flow rate of 1 mL /min .

35 Pass the fractions containing the rVapD through a HiPrep 26/10 Desalting at a flow rate of 1 mL /min to remove imidazole.

Note

If a desalting column is unavailable, the imidazole can be removed by dialysis.

36 Analyze the purified rVapD. Take 20 µL of the sample, add 2.5 µL of 4× BMR buffer, and boil it for 00:03:00 . 3m

37 Separate the sample by SDS-PAGE.

38 Stain the gel with Coomassie brilliant blue solution.

## Production of polyclonal antibodies against VapD

39 A total of six six-week-old Balb/C mice were used. The mice were intraperitoneally immunized five times, fortnightly, with 10 µg of rVapD diluted in PBS 1× and mixed with Freund's incomplete adjuvant, in a total volume of 200 µL per animal. Four serum samples were collected. After the final sample collection, the mice were euthanized with $CO_2$. The experiment is depicted in Fig 1.

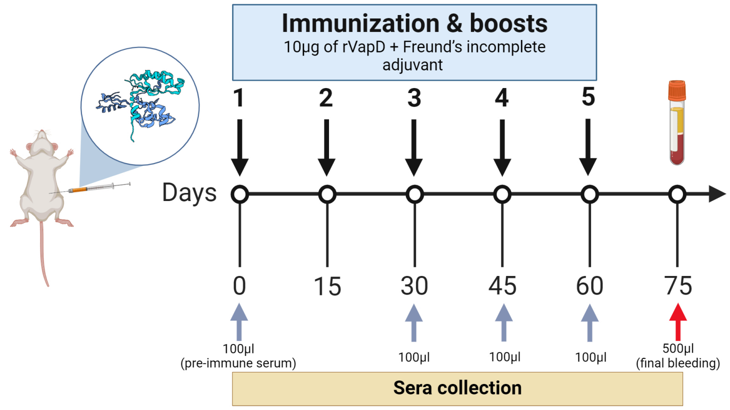


**Fig 1.** Representation of the immunization schedule. Sera were collected for antibody analysis at specific time point: before the first inoculation (pre-immune serum) and at 15, 45, and 60 days after each booster doce (gray arrows). The final blood collection was performed on day 75 (red arrow). This figure was created with **https://www.biorender.com/**

40 Dilute rVapD in carbonate-bicarbonate buffer a final concentration of 100 ng /mL .

41 Coat a flat-bottomed 96-well high binding plate with 100 µL /well of rVapD diluted in carbonate buffer.

42 Incubate Overnight at 4 °C . 8h

43 Wash three times 200 µL /well of PBT.

44 Block the unoccupied sites with 200 µL /well of 5% skimmed milk diluted in PBT.

45 Incubate for 00:40:00 at 37 °C . 40m

46 Incubate the plates for 02:00:00 at 37 °C with 100 µL /well of serum from immunized mice, prepared in serial two-fold dilutions ranging from 1:500 to 1:32,000. 2h

47 Wash three times with 200 µL /well of PBT.

48 Incubate the plate with the secondary antibody mouse antibody H+L coupled to horseradish peroxidase for 01:30:00 at 37 °C . 1h 30m

49 Wash three times with 200 µL /well of PBT.

50 Add 100 µL /well of the OPD developer solution.

51 Incubate for 00:20:00 at Room temperature . 20m

52 Stop the reaction with 100 µL /well of [M] 3 Molarity (M) sulfuric acid.

53 Read at 492nm in a spectrophotometer.

54 For the dot blot, fix a nitrocellulose membrane with different concentrations of rVapD Overnight at 4 °C . The initial concentration is 1 µg /mL of rVapD, serially diluted two-fold down to 0.03 µg /mL . 8h

55 Block the membranes with 5% skimmed milk diluted in PBT and incubate for 00:40:00 with gentle shaking at Room temperature . 40m

56 Wash the membrane three times with PBT.

57 Incubate with the mouse serum (anti-VapD) at dilution of 1:500, 1:1,000, and 1:2,000 for 02:00:00 with gentle shaking at Room temperature . 2h

58 Wash the membrane three times with PBT.

59 Incubate with the secondary antibody (anti-mouse antibody H+L coupled to horseradish peroxidase) at a 1:2,000 dilution for 02:00:00 with gentle shaking at Room temperature . 2h

60 Visualize the enzymatic reaction with the DAB developer solution and stop it with distilled water.

## Western blot anti-VapD 11h 20m

61 Load in 20% SDS-PAGE gels with different concentrations of rVapD, and run.

62 Transfer the SDS-PAGE gel to a PVDF membrane for 01:00:00 at a constant voltage of 100 V at 4 °C . 1h

63 Block the unoccupied sites with 3% skimmed milk in PBS 1× for 01:00:00 at Room temperature with gentle rotation. 1h

64 Incubate the membrane with mouse serum (anti-VapD) as the primary antibody in a dilution of 1:2,000 Overnight at 4 °C with gentle agitation. 8h

65 Wash three times with PBT for 00:10:00 . 10m

66 Incubate the membrane with the secondary antibody (anti-mouse antibody H+L coupled to horseradish peroxidase) at a 1:2,000 dilution for 01:00:00 at 4 °C with gentle agitation. 1h

67 Wash three times with PBT for 00:10:00 . 10m

68 Perform the protein detection using a chemiluminescence reagent and a blot scanner.

> **Note**
>
> If a blot scanner is unavailable, the chemiluminescence signal can be detected in a dark room using radiographic plaques.

## Detection of VapD in *Helicobacter pylori* and AGS cells co-cultures 4d 23h 26m

69 Grow Human gastric adenocarcinoma cell lines (AGS) in DMEM supplemented with 5% fetal bovine serum, 100 IU/mL penicillin, 100 mg /mL streptomycin, 0.2% NaHCO3, [M] 10 millimolar (mM) HEPES, and [M] 2 millimolar (mM) glutamine.

70 Incubate at 37 °C in humidified atmosphere, in the presence of 5% $CO_2$.

71 Grow *H. pylori* strain 26695 in blood agar plates supplemented with 5% fetal bovine serum.

72 Incubate at 37 °C for 48:00:00 in humidified atmosphere, in the presence of 8% $CO_2$. 2d

73 Seed $2.5x10^5$ AGS cells in 35 mm Petri dishes.

74 Incubate at 37 °C Overnight in humidified atmosphere, in the presence of 5% $CO_2$. 8h

75 Wash three times with PBS 1× and inoculate *H. pylori* strain 26695 at MOI of 100 (multiplicity of infection of 100 bacteria/cell) in 2.5 mL of DMEM.

76 Incubate at 37 °C for 03:00:00 in humidified atmosphere, in the presence of 5% $CO_2$. 3h

77 Remove the supernatant, and add 2.5 mL of DMEM containing 200 µg /mL gentamicin.

78 Incubate for an additional 02:00:00 . 2h

79 Wash three times with PBS 1×.

80 Add 2.5 mL of DMEM supplemented with 2% fetal bovine serum.

81 Place three coverslips in each Petri dish and incubate at 37 °C in a humidified atmosphere in the presence of 5% $CO_2$ for up to 48:00:00 . 2d

82 Wash the cells with PBS 1×.

83 Fix with fixative solution for 00:20:00 at Room temperature . 20m

84 Wash the cells with PBS 1× and permeabilize them with permeabilizing solution for 00:05:00 at 4 °C . 5m

85 Incubate the cells with mouse serum (anti- vapD) at a dilution of 1:100 in PBS 1× and anti-*H. pylori* at a dilution of 1:200, Overnight at 4 °C . 8h

86 Wash the cells with PBS 1×.

87 Incubate for 02:00:00 at Room temperature with anti-mouse Alexa Fluor 594 and anti-rabbit Alexa Fluor 488 antibodies diluted 1:100 in PBS 1×. 2h

88 Wash the cells with PBS 1× .

89 Incubate with DAPI (4´, 6-diamino-2-phenilindol) for 00:01:00 at Room temperature . 1m

90 Wash the cells with PBS 1X.

91 Mount with mounting medium for fluorescence (Antifade Mounting Medium).

92 Observe the samples under an epifluorescence microscope.

## Immunoprecipitation of VapD

4h 5m

93 Wash the protein A/G agarose beads ( 20 µL per reaction) two times with 200 µL of lysis buffer supplemented with EDTA-free protease inhibitor cocktail and [M] 0.2 millimolar (mM) PMSF.

94 Centrifuge at 500 x g, 00:01:00 . 1m

95 Resuspend the washed beads in five volumes of lysis buffer.

96 Incubate the beads with 5 µL of mouse serum (anti-VapD) diluted in lysis buffer for 02:00:00 at 4 °C with gentle agitation. 2h

97 Recover the beads by centrifugation at 500 x g, 4°C, 00:01:00 , and discarding the supernatant (unbound antibody). 1m

98 Wash the beads two times with 10 volumes of lysis buffer and maintain them at 4 °C .

99 Dilute 20 µL of lysate of Rosetta (DE3) overexpressing rVapD with lysis buffer to a final volume of 100 µL .

100 Incubate the antibody-coupled beads with the bacterial lysate for 02:00:00 at 4 °C with gentle agitation. 2h

101 Collect the immunoprecipitates by centrifugation at 500 x g, 4°C .

102 Wash three times with 100 µL of lysis buffer.

103 Elute the proteins complexes by adding 20 µL of 4× BMR buffer and boiling for 00:03:00 . 3m

104 Load the samples on 20% SDS-PAGE gels and run.

105 Stain the gel with Coomassie brilliant blue solution.


## Acknowledgements

We thank Jose Luis Mendez for helpful laboratory techniques.
