# Supplementary figures and images for: Effective polyclonal antibodies against the virulence-associated protein D (VapD) of Helicobacter pylori, obtained from recombinant VapD
Source: PLoS One. 2025 Apr 16;20(4):e0321455. doi: 10.1371/journal.pone.0321455 (PMC12002528; doi:10.1371/journal.pone.0321455)

Figure 2

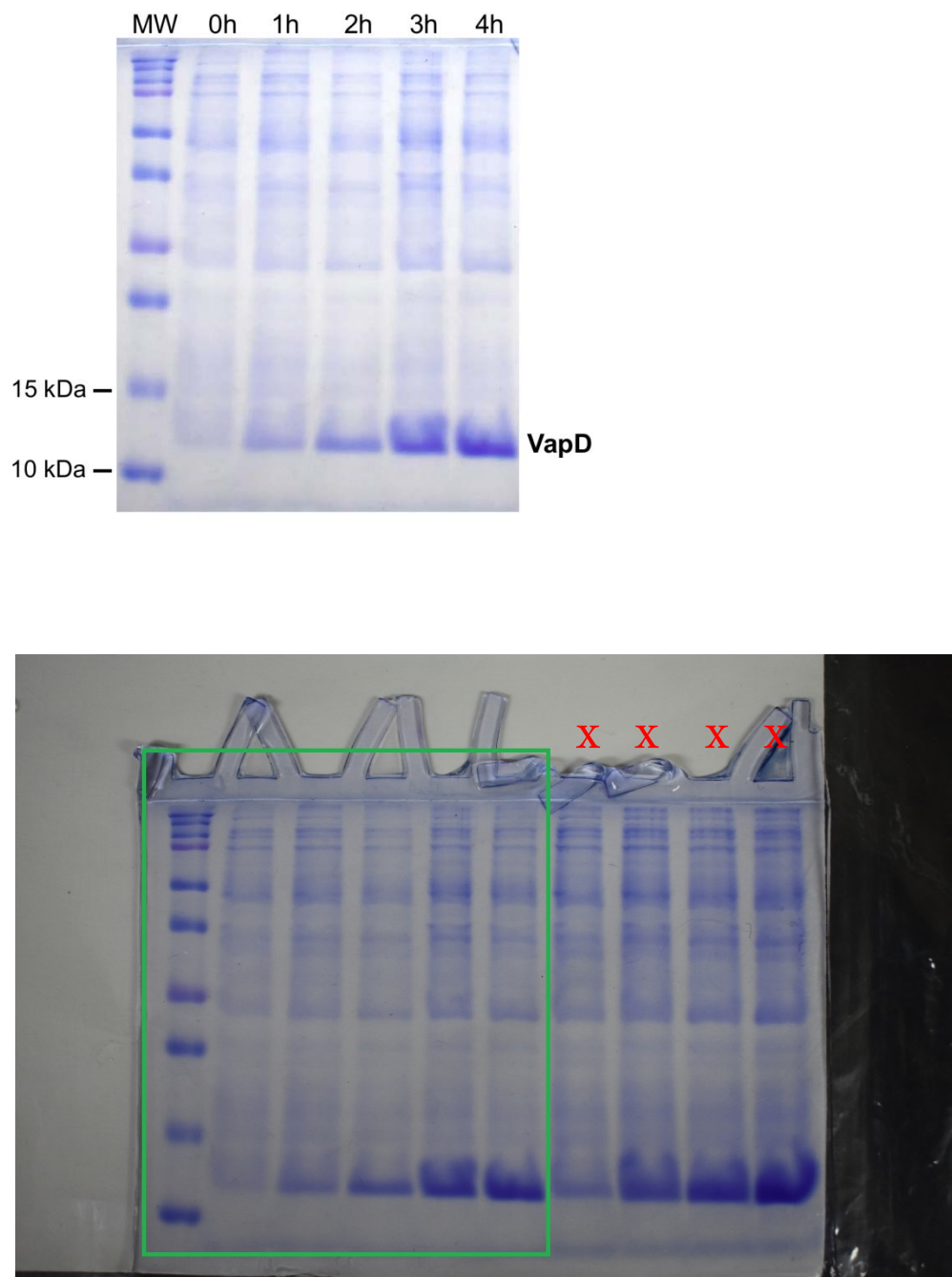

Figure 3

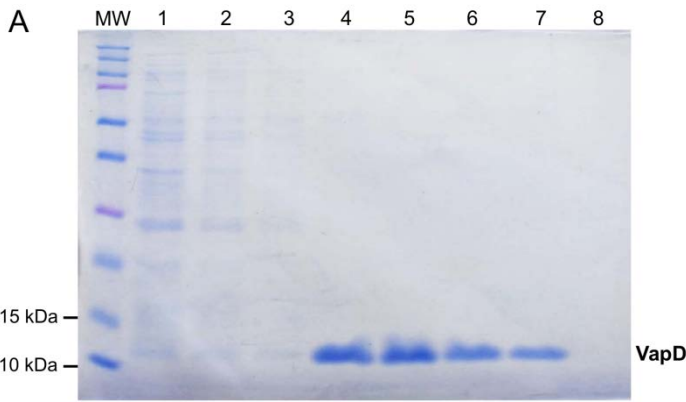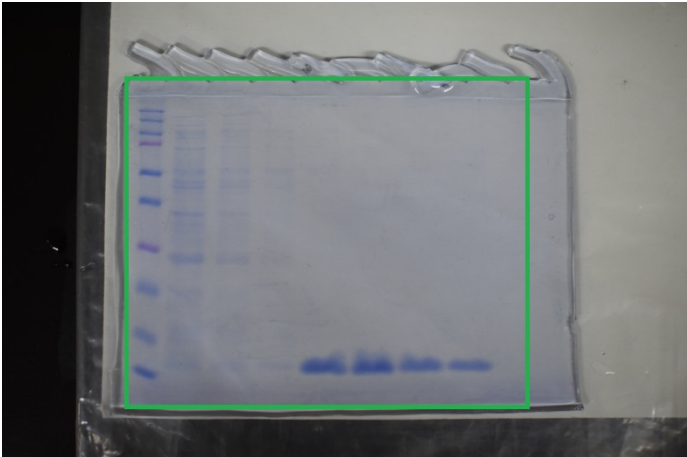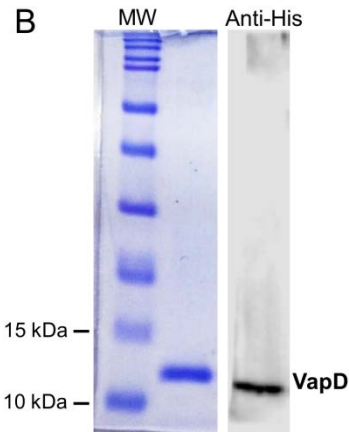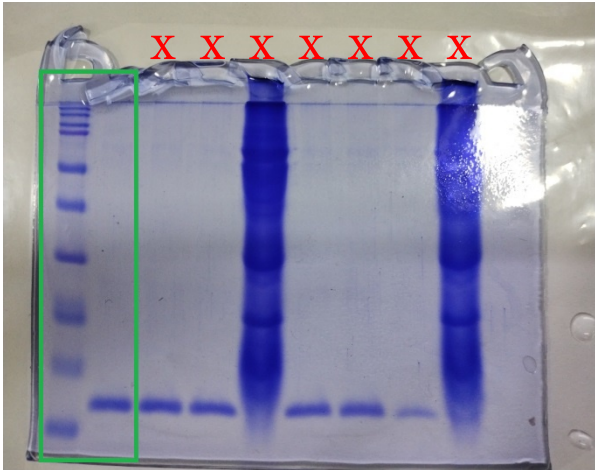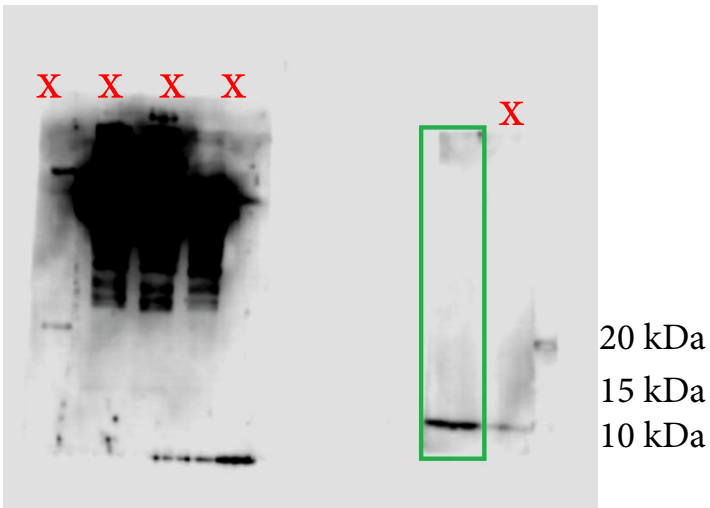

Figure 4

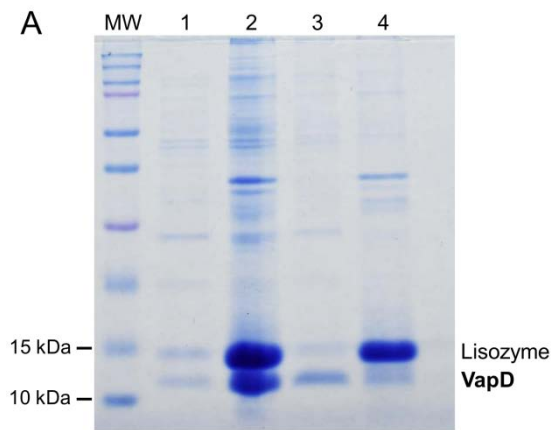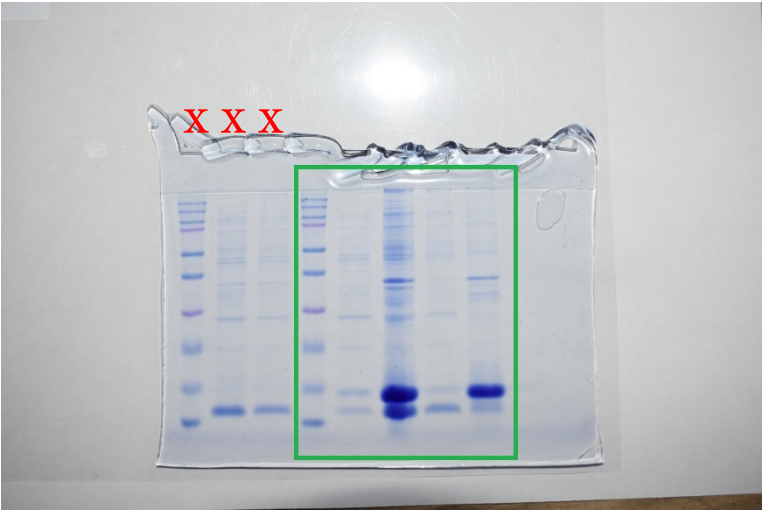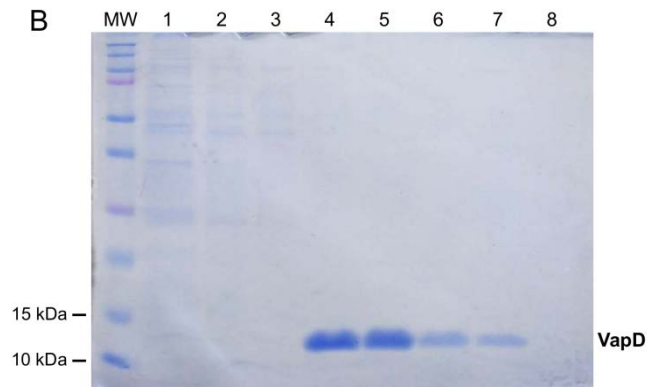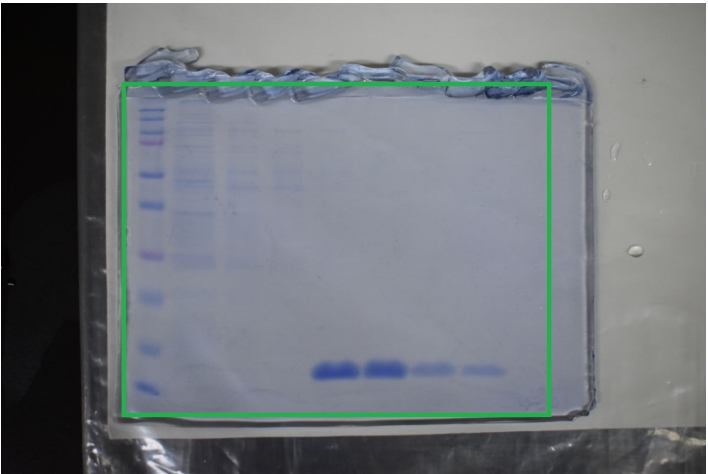

Figure 5

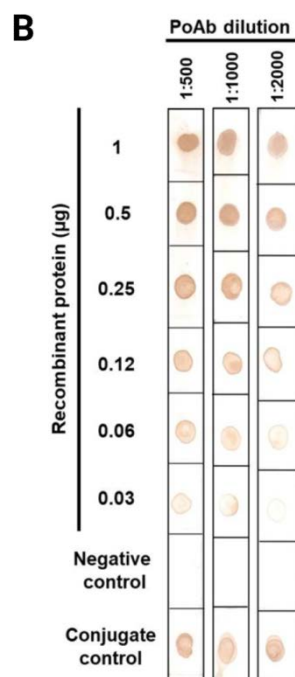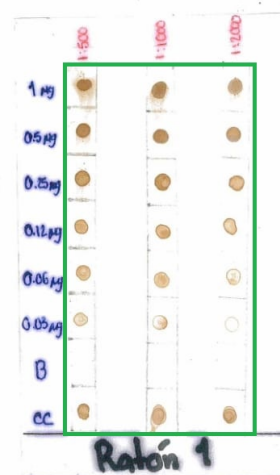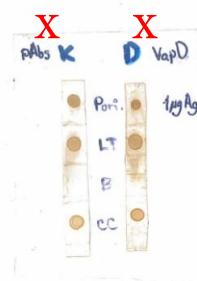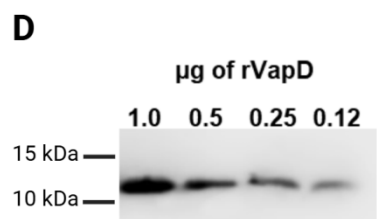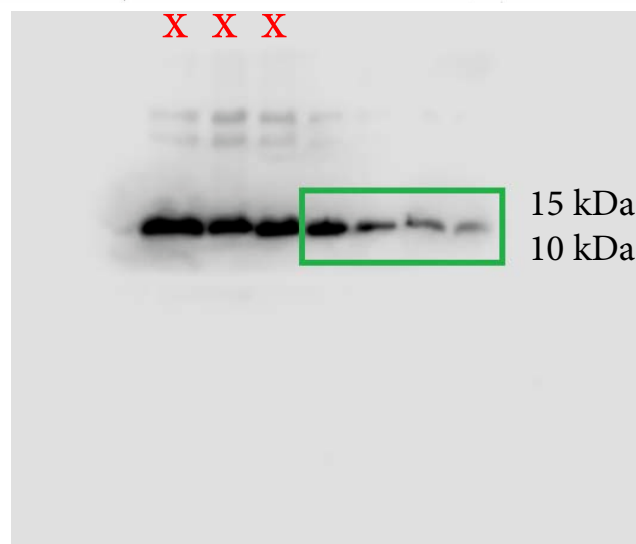

Figure 6

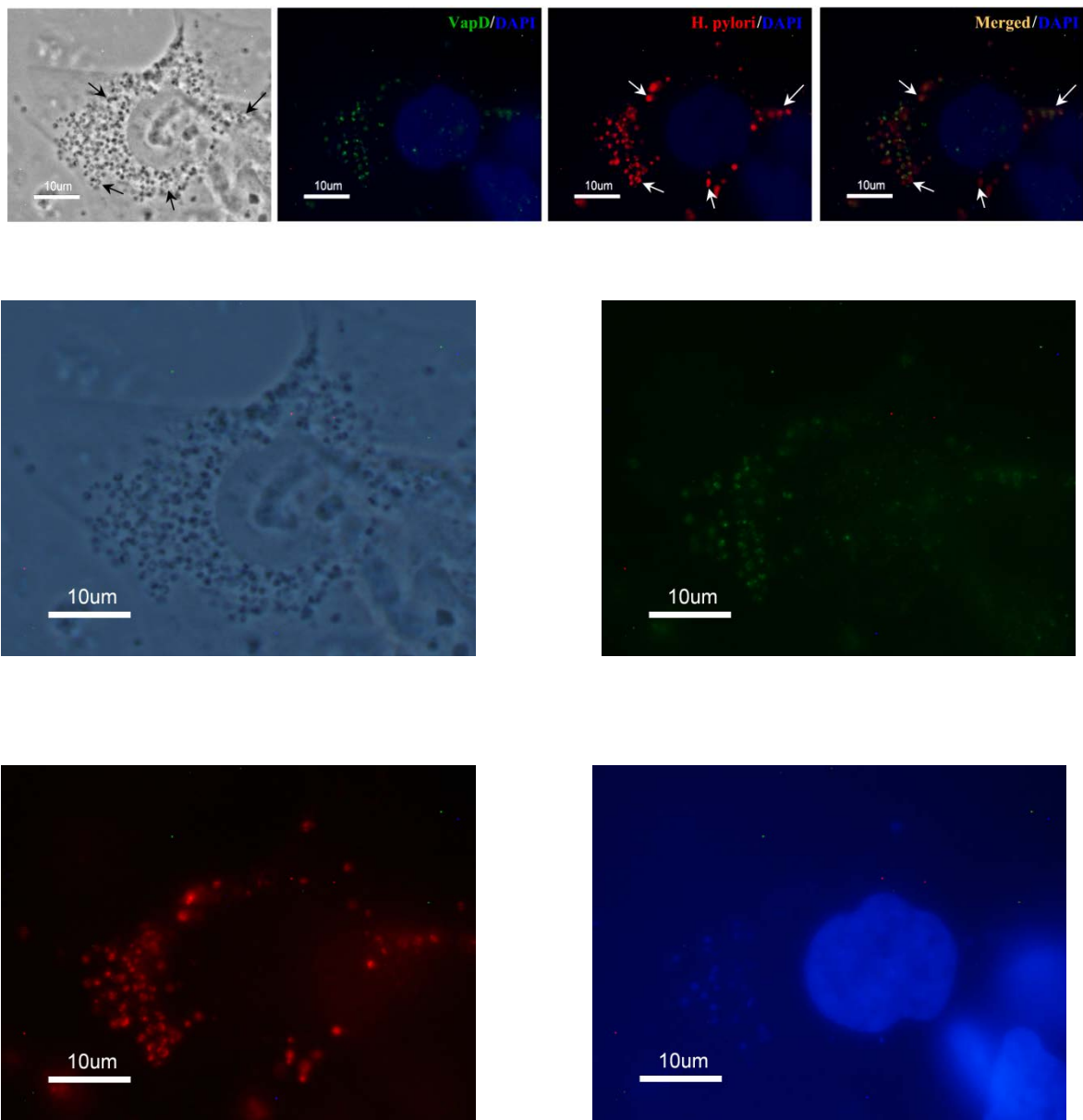

Figure 7

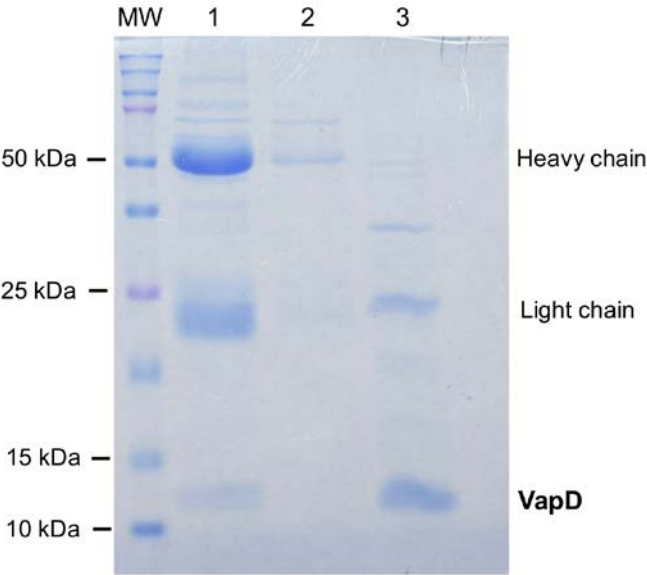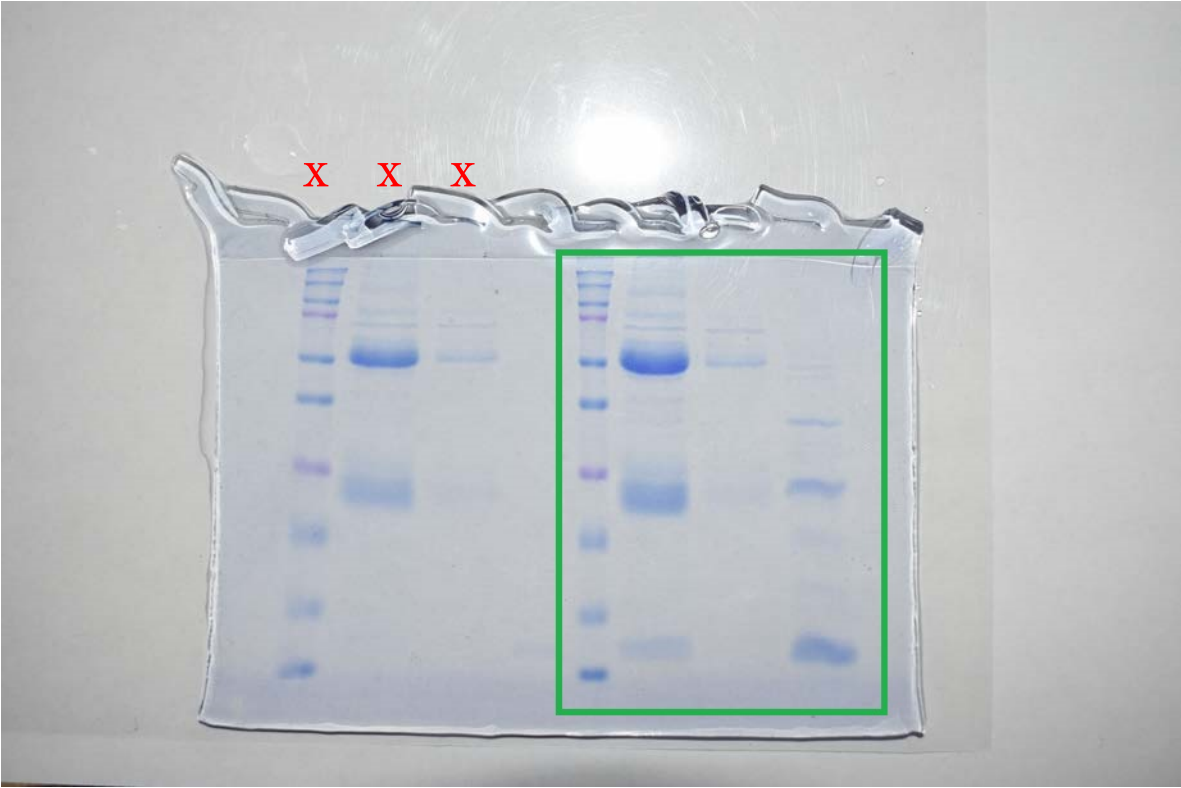

Supplement: S2 Raw Images — (PDF) [file pone.0321455.s002.pdf]
